# Large Margin and Local Structure Preservation Sparse Representation Classifier for Alzheimer’s Magnetic Resonance Imaging Classification

**DOI:** 10.3389/fnagi.2022.916020

**Published:** 2022-05-25

**Authors:** Runmin Liu, Guangjun Li, Ming Gao, Weiwei Cai, Xin Ning

**Affiliations:** ^1^College of Sports Engineering and Information Technology, Wuhan Sports University, Wuhan, China; ^2^College of Sports Science and Technology, Wuhan Sports University, Wuhan, China; ^3^School of Artificial Intelligence and Computer Science, Jiangnan University, Wuxi, China; ^4^AiTech Artificial Intelligence Research Institute, Changsha, China; ^5^Institute of Semiconductors, Chinese Academy of Sciences, Beijing, China

**Keywords:** Alzheimer’s disease, sparse representation classifier, image classification, magnetic resonance imaging, KAGGLE Alzheimer’s dataset

## Abstract

Alzheimer’s disease (AD) is a progressive dementia in which the brain shrinks as the disease progresses. The use of machine learning and brain magnetic resonance imaging (MRI) for the early diagnosis of AD has a high probability of clinical value and social significance. Sparse representation classifier (SRC) is widely used in MRI image classification. However, the traditional SRC only considers the reconstruction error and classification error of the dictionary, and does not consider the global and local structural information between images, which results in unsatisfactory classification performance. Therefore, a large margin and local structure preservation sparse representation classifier (LMLS-SRC) is developed in this manuscript. The LMLS-SRC algorithm uses the classification large margin term based on the representation coefficient, which results in compactness between representation coefficients of the same class and a large margin between representation coefficients of different classes. The LMLS-SRC algorithm uses local structure preservation term to inherit the manifold structure of the original data. In addition, the LMLS-SRC algorithm imposes the ℓ_*2,1*_-norm on the representation coefficients to enhance the sparsity and robustness of the model. Experiments on the KAGGLE Alzheimer’s dataset show that the LMLS-SRC algorithm can effectively diagnose non AD, moderate AD, mild AD, and very mild AD.

## Introduction

Alzheimer’s disease (AD) is a chronic progressive neurodegenerative disease that usually progresses slowly in the early stages and gets worse over time (Katabathula et al., 2021). AD often occurs in the elderly. The initial symptoms are easy to forget recent events. With the development of the disease, the symptoms may include language problems, disorientation, mood swings, loss of self-care ability, etc., which will eventually seriously affect the daily life of the elderly. Currently, about 90 million people worldwide suffer from AD of varying degrees. It is estimated that by 2050, the number of AD patients will reach 300 million (Wong, 2020). The specific symptoms of very mild AD are progressive decline in memory or other cognitive functions, but do not affect the ability of daily living. According to statistics, about 10–15% of very mild AD will eventually transform into AD (Porsteinsson et al., 2021). Current scientific and clinical research has not yet clearly identified the pathogenesis and etiology of AD, and there is no fully effective treatment drug. AD is uncontrollable and irreversible after being diagnosed. However, if patients can be intervened and treated in the early stage of mild cognitive impairment (MCI), it is hoped that the onset of AD will be delayed by 5 years, and even stop the progression of AD in the stage of MCI, and no longer worsen into AD, reducing the number of patients with AD by 40% (Venugopalan et al., 2021).

In the past decade, neuroimaging techniques have been widely used in the classification and prediction of AD. Among them, magnetic resonance imaging (MRI) is a non-contact imaging technology that can provide detailed three-dimensional anatomical images of the brain and provide effective information for the classification and prediction of AD (Al-Khuzaie and Duru, 2021). The AD classification algorithms based on machine learning usually extract the required features from the collected medical images by manual or semi-manual methods. Various parts of the brain regions of AD patients will atrophy to varying degrees due to the progression of the disease process. The volume, shape and texture information of the hippocampus, gray matter, white matter, and cerebral cortex of the brain are important features to distinguish AD and healthy people (Lee et al., 2020; Gao, 2021). To classify AD MRI images, some studies extract the volume information of the whole brain or part of the brain. Some scholars segment different regions of the brain and take the volume of each segment as features. According to the anatomical automatic labeling brain region template, some researchers divide the entire brain or part of the brain region into multiple regions and then obtain the features for each region. AD Patients often experience cerebral cortex atrophy and ventricular enlargement, and early AD patients usually have hippocampal atrophy (van Oostveen and de Lange, 2021). Therefore, some scholars use the volume information of different regions of interest such as the hippocampus as features based on medical prior knowledge. Another common feature extraction method is the morphometric measurement method, which is often implemented based on MRI images and PET images. For example, Al-Khuzaie and Duru (2021) took the overall shape of the brain in MRI images as features. Katabathula et al. (2021) used the shape information of the hippocampus as features. Brain gully dilation is often seen in AD patients. Furthermore, texture features are also widely used in MRI images. Gao (2021) extracted the grayscale co-occurrence matrix of images as features. Hett et al. (2018) used 3D Gabor filter to extract and classify multi-directional texture features of MRI images.

Classifiers such as sparse representation classifier (SRC), logistic regression (LR), support vector machine (SVM), and decision tree (DT) are widely used in AD MRI image classification. For example, Kruthika et al. (2019) used a multi-level classifier to classify AD MRI images. They first used a naive Bayes classifier, and then used SVM as secondary classification to classify the data with confidence lower than the threshold. Liu et al. (2015) proposed a multi-view learning algorithm based on inherent structure of mild cognitive impairment (MCI) MRI images, which used the multi-view features of MCI images to train multiple SVMs, and then fused and discriminated each classifier result. Altaf et al. (2018) used SVM, random forest, and *K*-nearest neighbor (KNN) to train AD classifiers, respectively, and the final classification result was the weighted sum of the results of each classifier. Yao et al. (2018) used the idea of hierarchical classification to classify AD MRI images. They initially classified samples into four classes (AD, healthy, MCI, converted MCI), then they trained several binary classifiers (AD and converted MCI, healthy and MCI), and finally got a classifier that can classify all samples into four classes. Pan et al. (2019) proposed an algorithm to integrate multi-level features based on FDG-PET images, and simultaneously considered the region features and connectivity between regions to classify AD or MCI from healthy people. Finally, multiple SVMs were used for voting classification, and good results had been achieved in multiple binary classification tasks.

Magnetic resonance imaging image features usually suffer from high dimensionality and small sample size, which may lead to overfitting in data-driven machine learning methods (Jiang et al., 2019). To solve this problem, most existing methods adopt feature selection or feature representation to exploit the potential knowledge of data. Sparse representation is one of the widely used feature representation methods. Sparse representation can explore potential relationships within the data (Gu et al., 2021). Chang et al. (2015) proposed a dictionary learning algorithm based on sparse decomposition of stacked prediction. They used the spatial pyramid matching method to encode representation coefficients, and used SVM to classify the pathological state of tumors. Shi et al. (2013) developed a multi-modal SRC algorithm for lung histopathological image classification, which used genetic algorithm to guide the learning of three sub-dictionaries of color, shape and texture, and then combined sparse reconstruction error and majority voting algorithm for classification of lung histopathology images. He (2019) proposed a spatial pyramid matching algorithm based on joint representation coefficient, which utilized the three color channel information of RGB, and converted the grayscale description operator into a color description operator, which improved the image classification performance. Jiang et al. (2019) extracted features from breast cancer histopathological images based on stacked sparse autoencoder, and used Softmax function to detect cell nuclei in histopathological images. Zhang et al. (2016) realized the fusion of global and local features of the nuclear image, and then combined the ranking and majority voting algorithm to classify the histopathological images of breast cancer. The above algorithms can effectively extract image features by introducing the sparsity of the image, and the extracted features have good reconstruction properties, but they do not have good discriminative ability.

To improve the diagnosis of MCI and AD based on MRI images, we propose large margin and local structure preservation sparse representation classifier (LMLS-SRC) in this manuscript. The traditional SRC only uses the classification error term to control the classification accuracy, and does not fully consider the class label information of the representation coefficients. Different from the traditional SRC, the LMLS-SRC algorithm introduces the classification margin term of representation coefficients into the sparse representation classifier, so that the similar representation coefficients are compact in the representation space, and the dissimilar representation coefficients are separated as much as possible in the representation space. Experiments on the KAGGLE Alzheimer’s dataset verify the advantages of our algorithm. Major contributions of this manuscript are highlighted below: (1) Considering the global information of the data by using the large margin term, the obtained dictionary is discriminative, and the representation coefficient has the small intra-class distance and large inter-class distance. (2) The local structure preservation term is introduced, which can inherit the manifold structure of the original data. (3) The ℓ_*2,1*_-norm term on the representation coefficients is used, which can enhance the sparsity and robustness of the representation coefficients.

## Backgrounds

### Dictionary-Based Sparse Representation Classifier

Using SRC algorithm in image classification, how to design effective dictionary and representation coefficient for feature representation is the key factor to determine the algorithm performance (Wright et al., 2009). There are three aspects considered in the design of SRC algorithm: (1) The reconstruction error of the representation coefficients is small, so that the samples are as close to the original samples as possible in the sparse representation; (2) The representation coefficients are constrained to make the representation coefficients as sparse as possible; (3) The discrimination term should be considered to better extract more discriminative information of data (Jiang et al., 2013).

Let **X** = [**X**_1_,…,**X**_*K*_] ∈ **R**^*d*×*N*^ be the *K*-classes training sample set, **X**_*k*_ = [**x**_1_,…,**x**_*N*_*k*__] be the *k*-th class training sample subset, *k* = 1, 2,…, *K*,*N* = *N*_1_ + *N*_2_ + ⋯ + *N*_*K*_. *d* is the dimensional of samples. The SRC algorithm for image classification can be represented as,


(1) $$\min_{D,A}{||\text{X-DA}||}_{F}^{2}+\lambda \,g\,\left(A\right)+\eta \,f\,\left(D,A,Y\right),$$

where **Y** is the class label matrix of **X**. **D** ∈ **R**^*d*×*m*^ is the learned dictionary, and **A** ∈ **R**^*m*×*N*^ is the representation coefficient matrix of **X**. *m* is the size of dictionary. In model training, the data reconstruction item ${||\text{X-DA}||}_{F}^{2}$ is to ensure the representation ability of the dictionary **D**, so that the reconstruction error of the training data is minimized, and the reconstructed image is as close to the original sample as possible. The regularization term is used to constrain the sparsity of the representation coefficients, which is usually represented as,


(2) $$g\,\left(A\right)={||A||}_{p}.$$

where ||⋅||_*p*_ is the regularization term of the representation coefficient **A** (*p* < 2), which makes the representation coefficient as sparse as possible. *f*(**D**,**A**,**Y**) is the discriminative function term of representation coefficient for classification to ensure the discriminative ability of **D** and **A**.

To obtain a discriminative dictionary, Yang et al. (2017) developed a supervised Fisher discrimination dictionary learning (FDDL), which associated the elements in the dictionary with the class labels of the samples based on the Fisher discrimination criterion. Jiang et al. (2013) proposed the discriminative Label consistent K-SVD (LC-KSVD) algorithm. Zhang et al. (2019) proposed a robust flexible discriminative dictionary learning (RFDDL) algorithm based on subspace recovery and enhanced locality. This algorithm improved image representation and classification by enhancing representation coefficient robustness. The computational complexity of the SRC representation coefficient is usually high. To quickly obtain the representation coefficients, Ma et al. (2017) proposed the local sparse representation algorithm, which used the KNN criterion to select *k* samples adjacent to the current sample to build a dictionary matrix. In this way, the size of the dictionary is reduced and the process of representation coefficient is greatly accelerated. Similarly, inspired by the KNN criterion, Zheng and Ding (2020) developed a sparse KNN classifier based on group lasso strategy and KSVD algorithm. Wang et al. (2018) proposed a SRC algorithm based on the ℓ_*2*_-norm, which replaced the ℓ_*1*_-norm with the ℓ_*2*_-norm to constrain the coefficients. Ortiz and Becker (2014) proposed an approximate linear SRC algorithm. Authors used least square algorithm to select the training samples corresponding to the absolute values of the *k* largest coefficients to build a sub-dictionary.

### KAGGLE Alzheimer’s Image Dataset

**FIGURE 1:**
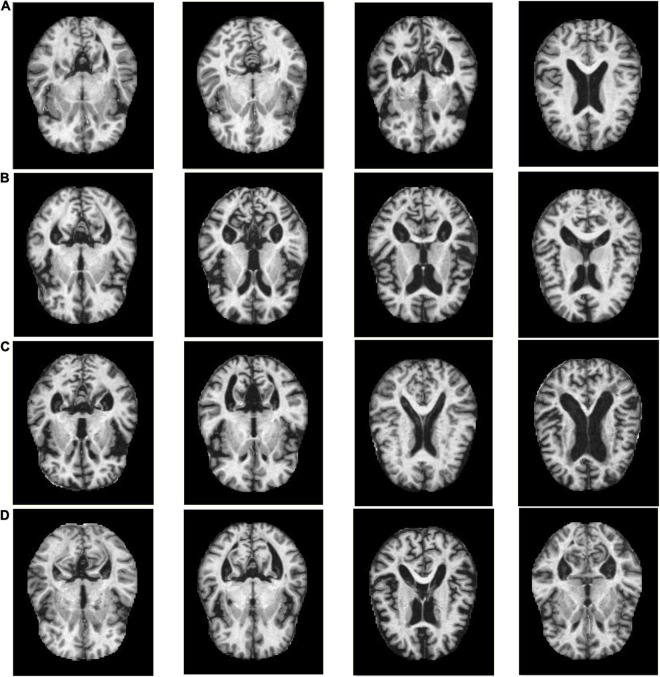
Example samples of the KAGGLE Alzheimer’s dataset, **(A)** Non AD, **(B)** Moderate AD, **(C)** Mild AD, **(D)** Very Mild AD.

The experiments in this manuscript are carried out on the KAGGLE Alzheimer’s image dataset (Loddo et al., 2022). The KAGGLE Alzheimer’s dataset contains a total of four types of MRI images: non AD (3,200 images), very mild AD (2,240 images), mild AD (896 images) and moderate AD (64 images), with the resolution of 176 × 208. The KAGGLE Alzheimer’s dataset does not provide detailed information on patient status. Figure 1 shows some example images of the KAGGLE Alzheimer’s dataset.

## The Proposed Algorihtm

### Objective Function

The purpose of sparse representation is to represent the sample with as few elements as possible on a given dictionary, so that a more concise representation of the sample can be obtained, and the useful information contained in the sample can be easily obtained. Thus the core problem of sparse representation is how to compute sparse coding coefficients on a given learned dictionary. Compared with the commonly used ℓ_*1*_-norm and ℓ_*2*_-norm, ℓ_*2,1*_-norm can improve the robustness of the model and reduce the computational complexity. Thus, we introduce ℓ_*2,1*_-norm constraint on representation coefficients in LMLS-SRC, i.e.,


(3) $$\Pi_{1}=\arg\,\min_{D,A}\left\{{||\text{X-DA}||}_{F}^{2}+\lambda_{1}\,{||A||}_{2,1}^{2}\right\},$$

where λ_*1*_ is a constant.

We define a large margin term on representation coefficient that relies on a specific neighborhood size for intra-class and inter-class representation coefficients. The large margin term minimizes the intra-class distance of the representation coefficient and maximizes the inter-class distance of the representation coefficient, so as to improve the difference between the representation coefficients of different classes. The large margin term on representation coefficient can be written as,


(4) $$f\,\left({\text{a}}_{i}\right)=\arg\min\left\{\sum_{t\in C_{k}}\frac{{||{\text{a}}_{i}-{\text{a}}_{t}||}^{2}}{N_{k}}-\sum_{j\notin C_{k}}\frac{{||{\text{a}}_{i}-{\text{a}}_{j}||}^{2}}{N-N_{k}}\right\},$$

where $\sum_{t\in C_{k}}\frac{{||a_{i}-a_{t}||}^{2}}{N_{k}}$ represents the distance between *a*_*i*_ and the sparse representation of the same class. $\sum_{j\notin C_{k}}\frac{{||a_{i}-a_{t}||}^{2}}{N-N_{k}}$ represents the distance between *a*_*i*_ and the sparse representation of the different class. *C*_*k*_ is the index set of the *k*-th class sample.

We build the intra-class similarity matrix *Q^w^* and inter-class similarity matrix *Q^b^* based on representation coefficient. The elements of the matrix *Q^w^* and matrix *Q^b^* are expressed as,


(5) $$q_{i\,j}^{w}=\left\{ \begin{array}{c} 1/N_{k},i\,f\,i,j\in C_{k}\\ 0,o\,t\,h\,e\,r\,w\,i\,s\,e \end{array}\right.$$

(6) $$q_{i\,j}^{b}=\left\{ \begin{array}{c} 1/\left(N-N_{k}\right),i\,f\,i\in C_{k},j\notin C_{k}\\ 0,o\,t\,h\,e\,r\,w\,i\,s\,e \end{array}\right.$$

Then the large margin term on representation coefficient can be expressed as,


(7) $$\begin{array}{c} \Pi_{2}=\arg\,\min_{A}\frac{1}{N}\,\sum_{i=1}^{N}f\,\left({\text{a}}_{i}\right)\\ =\frac{1}{N}\,\sum_{i=1}^{N}\sum_{j=1}^{N}\left(q_{i\,j}^{w}\,{||{\text{a}}_{i}-{\text{a}}_{j}||}^{2}-q_{i\,j}^{b}\,{||{\text{a}}_{i}-{\text{a}}_{j}||}^{2}\right)\\ =\frac{1}{N}\,\left(2\,\sum_{i=1}^{N}{\text{a}}_{i}^{2}-2\,\sum_{i=1}^{N}\sum_{j=1}^{N}{\text{a}}_{i}\,q_{i\,j}^{w}\,{\text{a}}_{j}\right)-\\ \frac{1}{N}\,\left(\sum_{i=1}^{N}{\text{a}}_{i}^{2}+\sum_{j=1}^{N}{\text{a}}_{j}^{2}\,q_{j\,j}^{b}-2\,\sum_{i=1}^{N}\sum_{j=1}^{N}{\text{a}}_{i}\,q_{i\,j}^{b}\,{\text{a}}_{j}\right)\\ =t\,r\,\left(\frac{1}{N}\,{\text{A}}^{T}\,\left(2\,\text{I}-2\,{\text{Q}}^{w}\right)\,\text{A}\right)-t\,r\,\left(\frac{1}{N}\,{\text{A}}^{T}\,\left(\text{I}+{\tilde{\text{Q}}}^{b}-2\,{\text{Q}}^{b}\right)\,\text{A}\right)\\ =t\,r\,\left(\frac{1}{N}\,{\text{A}}^{T}\,\left(\text{I}-2\,{\text{Q}}^{w}-{\tilde{\text{Q}}}^{b}+2\,{\text{Q}}^{b}\right)\,\text{A}\right)\\ =t\,r\,\left({\text{A}}^{T}\,\text{SA}\right) \end{array}$$

where $\text{S}=\frac{1}{N}\,\left(\text{I}-2\,{\text{Q}}^{w}-{\tilde{\text{Q}}}^{b}+2\,{\text{Q}}^{b}\right)$. The matrix ${\tilde{\text{Q}}}^{b}$ is the diagonal matrix with the element being the column-sum of **Q**^*b*^.

Following the principle of local structure preservation, if two images are close in the original space, they should also have similar representation coefficients. To this end, we construct a similarity matrix **P** that reflects the intrinsic local structure between images. The element of matrix **P** is defined as,


(8) $$p_{i\,j}=\left\{ \begin{array}{c} \exp\left(-\frac{{||x_{i}-x_{j}||}_{2}^{2}}{2\,\sigma^{2}}\right),i\,f\,{\text{x}}_{i}\in N\,\left({\text{x}}_{j}\right)\,o\,r\,{\text{x}}_{j}\in N\,\left({\text{x}}_{i}\right),\\ 0,o\,t\,h\,e\,r\,w\,i\,s\,e, \end{array}\right.$$

where *N*(**x**_*j*_) represents the *k* nearest neighbors of **x**_*j*_.

The local structure preservation term on representation coefficient is expressed as,


(9) $$\begin{array}{c} \Pi_{3}=\arg\,\min_{A}\sum_{i,j}^{N}p_{i\,j}\,{||{\text{a}}_{i}-{\text{a}}_{j}||}_{2}^{2}\\ =t\,r\,\left({\text{A}}^{T}\,\left(\text{P}-\tilde{\text{P}}\right)\,\text{A}\right)\\ =t\,r\,\left({\text{A}}^{T}\,\text{LA}\right), \end{array}$$

where the graph Laplacian matrix **L** is $\text{L}=\text{P}-\tilde{\text{P}}$,$\tilde{\text{P}}$ is the diagonal matrix with the element being the row-sum of **P**.

The LMLS-SRC algorithm is a supervised learning model. Using the class labels of all training samples, we use a linear classifier **W** for representation coefficient **A** and dictionary **D**, i.e.,


(10) $$\Pi_{4}=\arg\min_{W,A}||\text{WA}-\text{Y}{||}_{F}^{2}+\lambda_{5}||\text{W}{||}_{F}^{2}.$$

In summary, the objective function of the LMLS-SRC algorithm can be written as,


(11) $$F\,\left(D,A,W\right)=\min_{D,A,W}\Pi_{1}+\Pi_{2}+\Pi_{3}+\Pi_{4},$$

i.e.,


(12) $$\begin{array}{c} \min_{D,A,W}{||\text{X–DA}||}_{F}^{2}+\lambda_{1}\,{||A||}_{2,1}^{2}+\lambda_{2}\,t\,r\,\left({\text{A}}^{T}\,\text{SA}\right)\\ +\lambda_{3}tr\left({\text{A}}^{T}\text{LA}\right)+\lambda_{4}||\text{WA}-\text{Y}{||}_{F}^{2}+\lambda_{5}||\text{W}{||}_{F}^{2},\\ s.t.{||d_{i}||}_{2}^{2}\leq 1,\forall i \end{array}$$

where λ_1_,λ_2_,λ_3_,λ_4_, and λ_5_ are trade-off parameters.

By alternately optimizing the representation coefficient **A**, dictionary **D** and classifier parameter **W**, the following performance can be obtained as: (1) the dictionary **D** has more sparse representation performance, which enhances the reconstruction of the sample by the dictionary. (2) LMLS-SRC maximizes the distance between different classes of representation coefficients and greatly reduces the similarity between different classes of representation coefficients. (3) The representation coefficient is more discriminative, which is beneficial to the performance of image classification.

### Optimization

(1) Fix **D**, **W**, and update **A**. Eq. (12) can be written by,


(13) $$\min F\,\left(A\right)={||\text{X–DA}||}_{F}^{2}+\lambda_{1}\,{||A||}_{2,1}^{2}+\lambda_{2}\,t\,r\,\left({\text{A}}^{\text{T}}\,\text{SA}\right)+\lambda_{3}tr\left({\text{A}}^{\text{T}}\text{LA}\right)+\lambda_{4}||\text{WA}-\text{Y}{||}_{F}^{2}.$$

According to the definition of ℓ_*2,1*_-norm, ${||A||}_{2,1}^{2}=t\,r\,\left({\text{A}}^{\text{T}}\,\Omega \,\text{A}\right)$. Ω is a diagonal matrix whose elements are setting by Ω_*ii*_ = 1/(2||**A**_*i*_||_2_) where **A**_*i*_ represents the *i*-th row of **A**.

Equation (12) can be re-written by,


(14) $$\min F\,\left(A\right)={||\text{X–DA}||}_{F}^{2}+\lambda_{1}\,t\,r\,\left({\text{A}}^{\text{T}}\,\Omega \,\text{A}\right)+\lambda_{2}\,t\,r\,\left({\text{A}}^{\text{T}}\,\text{SA}\right)+\lambda_{3}tr\left({\text{A}}^{\text{T}}\text{LA}\right)+\lambda_{4}||\text{WA}-\text{Y}{||}_{F}^{2}.$$

Setting ∂⁡*F*(**A**)/∂⁡**A** = 0, we can obtain,


(15) $$\frac{\partial L}{\partial A}=2\,{\text{D}}^{\text{T}}\,\text{D}\,A-2\,{\text{D}}^{\text{T}}\,\text{X}+\left(2\,\lambda_{1}\,\Lambda +2\,\lambda_{2}\,\text{S}+2\,\lambda_{3}\,\text{L}\right)\,\text{A}+2\lambda_{4}\left({\text{W}}^{\text{T}}\text{WA}-{\text{W}}^{\text{T}}\text{Y}\right).$$

**A** can obtained by the updated by,


(16) $${\text{A}}^{{}^{*}}={\left({\text{D}}^{\text{T}}\,\text{D}+\lambda_{1}\,\Lambda +\lambda_{4}\,{\text{W}}^{\text{T}}\,\text{W}+\lambda_{2}\,\text{S}+\lambda_{3}\,\text{L}\right)}^{-1}\left(\lambda_{4}\,{\text{W}}^{\text{T}}\,\text{Y}+{\text{D}}^{\text{T}}\,\text{X}\right).$$

(2) Fix **A**, **W**, and update **D**. Equation (12) can be written by,


(17) $$\begin{array}{c} \min F\,\left(D\right)={||\text{X–DA}||}_{F}^{2},\\ s.t.{||d_{i}||}_{2}^{2}\leq 1,\forall i \end{array}$$

We can solve Eq. (17) by the following Lagrangian dual function,


(18) $$\min F\,\left(D,\sigma \right)={||\text{X–DA}||}_{F}^{2}+\sum_{i=1}^{m}\gamma_{i}\,\left({||d_{i}||}_{2}^{2}-1\right),$$

where γ_*i*_ is the Lagrange multiplier of *i*-th atoms.

We build a diagonal matrix **Θ** with the element Θ_*ii*_ = γ_*i*_. Equation (18) can be written by,


(19) $$\min F\,\left(D,\Theta \right)={||\text{X–DA}||}_{F}^{2}+t\,r\,\left({\text{D}}^{\text{T}}\,\text{D}\,\Theta \right)-t\,r\,\left(\Theta \right).$$

Setting ∂⁡*F*(**D**,**Θ**)/∂⁡*D* = 0, we can obtain,


(20) $${\text{D}}^{{}^{*}}={\text{XA}}^{\text{T}}\,{\left({\text{AA}}^{\text{T}}+\Theta \right)}^{-1}.$$

(3) Fix **A** and **D**, and update **W**. Equation (12) can be written by,


(21) $$\min F\left(W\right)=\lambda_{4}||\text{WA}-\text{Y}{||}_{F}^{2}+\lambda_{5}||\text{W}{||}_{F}^{2}.$$

Setting ∂⁡*F*(**W**)/∂⁡*W* = 0, we can obtain,


(22) $${\text{W}}^{{}^{*}}=\lambda_{4}\,{\text{YA}}^{\text{T}}\,{\left(\lambda_{4}\,{\text{AA}}^{\text{T}}+\lambda_{5}\,\text{I}\right)}^{-1}.$$

**Table:** 

The optimization steps of LMLS-SRC algorithm are shown in Algorithm 1.
Input: training set **X** and its label matrix **Y**, tolerance error δ, maximum number of iterations *maxiter*, parameters λ_1_,λ_2_,λ_3_,λ_4_, and λ_5_, Output: parameters **D**, **A**, and **W**. Initialize: initialize **D** and **A** using the LC-KSVD algorithm, **W** = **I**, *m* = 1, Calculate matrices *Q^w^*, *Q^b^*, and **P**; While not converged and *m*≤*maxiter*do Calculate **D**(*m*) by Eq. (20); Calculate **A**(*m*) by Eq. (16); Calculate **W**(*m*) by Eq. (22); Check the convergence condition $\frac{\left|F\,\left(D\,\left(m\right),A\,\left(m\right),W\,\left(m\right)\right)-F\,\left(D\,\left(m-1\right),A\,\left(m-1\right),W\,\left(m-1\right)\right)\right|}{F\,\left(D\,\left(m-1\right),A\,\left(m-1\right),W\,\left(m-1\right)\right)}<\delta$ *m* = *m* + 1 end while

## Experiments

### Experimental Settings

In clinical diagnosis, AD classification tasks consist of two categories. The first is the AD binary classification task, which extracts features based on MRI images and uses machine learning models to classify normal individuals and AD patients, which can help doctors diagnose AD patients. The second is the classification of various ADs, especially the diagnosis and identification of mild AD and very mild AD. Early prediction of AD can help to take treatment and intervention measures in the early stage of AD. Therefore, in this manuscript, we design binary, three-class and four-class classification tasks on the KAGGLE Alzheimer’s dataset.

Volume analysis is the commonly used feature extraction method in AD classification. Volumetric feature extraction is divided into two categories: density maps and predefined area methods. AD MRI image is mainly related to the volume of the density map structure, cortical structure, subcortical structure and other regions. In this manuscript, we use FSL (FMRIB software library) toolbox to extract MRI features (Jenkinson et al., 2012). FSL is a library of comprehensive analysis tools for brain imaging data such as MRI, developed by the FMRIB Centre in Oxford. We use the FSL toolbox to calculate the volume, area and thickness characteristics of various brain tissues in brain MRI images. In the comparison experiment, the LMLS-SRC algorithm is compared with SRC (Wright et al., 2009), logistic regression (LR) (Tsangaratos and Ilia, 2016), linear discriminant (LD) (Kim et al., 2011), LC-KSVD, FDDL, and sparse representation-based discriminative metric learning (SRDML) (Zhou et al., 2022). The radial basis function (RBF) kernel is used in LR. The default settings are used to produce test results from these classifiers using the MATLAB classification learner toolbox. The RBF kernel and the regularization parameters for all comparison algorithms range from 10^–3^ to 10^3^. The number of dictionary atoms in SRC and dictionary learning is set as the number of training samples. Indicators of classification performance include classification accuracy, sensitivity, specificity, precision, F1-score, and G-mean. We carry out 5-fold cross-validation strategy and record the experimental results.

### Experimental Results

(1) Binary classification task. The main goal of this work is to classify brain MRI into AD and non AD classes. We utilized 3,200 and 62 MRI images for non AD and AD classes, respectively. We randomly selected 1,000 MRI images from the non AD class images to increase the moderate AD class dataset to 620 MRI images using data augmentation techniques. The comparative training and test results in binary classification task are shown in Tables 1, 2, respectively.

**TABLE 1:** The comparative training results (with standard deviation) in binary classification task.

Algorithms	Accuracy	Sensitivity	Specificity	Precision	F1-score	G-mean
LD	81.30	81.99	80.06	80.58	82.26	81.02
	(2.84)	(3.15)	(2.80)	(3.32)	(3.27)	(2.97)
LR	82.15	82.62	81.79	82.68	82.51	82.20
	(2.55)	(2.66)	(2.70)	(2.35)	(2.56)	(2.60)
SRC	82.10	78.97	77.33	77.63	77.55	78.15
	(2.35)	(2.01)	(2.64)	(1.62)	(1.43)	(2.28)
LC-KSVD	80.27	81.34	78.94	80.85	79.93	80.13
	(2.54)	(2.12)	(2.63)	(1.82)	(2.07)	(1.59)
FDDL	83.16	84.47	81.38	85.20	82.86	82.91
	(2.64)	(??)	(1.83)	(1.45)	(1.69)	(1.54)
SRDML	85.71	85.91	85.09	84.10	85.08	85.50
	(2.15)	(2.23)	(1.75)	(1.88)	(1.74)	(1.96)
LMLS-SRC	**89.80**	**90.39**	**87.87**	**88.89**	**90.43**	**89.12**
	(2.02)	(1.35)	(2.06)	(1.35)	(1.28)	(1.19)

*The bold values in Tables 1–6 are the best experiment results.*

**TABLE 2:** The comparative test results (with standard deviation) in binary classification task.

Algorithms	Accuracy	Sensitivity	Specificity	Precision	F1-score	G-mean
LD	80.92	81.64	80.44	81.45	80.70	81.04
	(2.26)	(1.69)	(2.10)	(2.06)	(1.62)	(1.37)
LR	81.61	82.28	80.96	82.81	80.79	81.62
	(1.71)	(2.58)	(2.70)	(1.04)	(1.88)	(2.64)
SRC	82.91	83.18	82.86	83.07	82.94	83.02
	(1.75)	(2.46)	(2.28)	(1.16)	(1.87)	(2.37)
LC-KSVD	82.15	82.59	80.51	82.78	82.56	81.54
	(2.74)	(1.38)	(2.80)	(2.55)	(1.96)	(1.93)
FDDL	82.89	84.26	81.71	84.35	83.23	82.98
	(2.23)	(1.50)	(1.43)	(1.14)	(2.02)	(1.46)
SRDML	85.44	87.13	84.35	86.42	85.42	85.73
	(2.14)	(2.20)	(2.10)	(2.74)	(2.05)	(2.15)
LMLS-SRC	**88.28**	**90.15**	**86.75**	**90.08**	**88.31**	**88.43**
	(2.07)	(2.06)	(1.67)	(1.92)	(1.18)	(1.68)

(2) Three-class classification tasks. The main goal of this work was to classify brain MRI into three classes: non AD, mild AD, and moderate AD. Using data augmentation techniques, these three classes of datasets contain 3,200, 700, and 620 images, respectively. We randomly selected 1,000 MRI images from the non AD class. The comparative training and test results in three-class classification task are shown in Tables 3, 4, respectively.

**TABLE 3:** The comparative training results (with standard deviation) in three-class classification task.

Algorithms	Accuracy	Sensitivity	Specificity	Precision	F1-score	G-mean
LD	80.13	80.70	80.52	79.57	80.94	80.61
	(2.72)	(1.92)	(2.24)	(2.28)	(2.34)	(2.36)
LR	81.31	82.55	80.25	81.17	81.82	81.39
	(2.55)	(2.30)	(2.03)	(2.62)	(2.19)	(2.16)
SRC	81.94	82.20	80.46	81.09	81.17	81.33
	(2.20)	(2.49)	(2.59)	(2.10)	(2.21)	(2.54)
LC-KSVD	83.80	85.54	81.64	83.94	83.32	83.57
	(1.76)	(1.68)	(2.98)	(2.23)	(1.80)	(2.24)
FDDL	84.04	86.12	81.13	83.93	84.32	83.59
	(2.30)	(2.61)	(2.33)	(2.24)	(2.36)	(2.47)
SRDML	85.39	86.82	84.88	86.32	86.86	85.85
	(2.33)	(2.00)	(2.37)	(2.05)	(2.33)	(2.02)
LMLS-SRC	**89.32**	**91.38**	**86.81**	**88.86**	**89.00**	**89.07**
	(1.84)	(1.20)	(2.81)	(2.12)	(1.53)	(1.83)

**TABLE 4:** The comparative test results (with standard deviation) in three-class classification task.

Algorithms	Accuracy	Sensitivity	Specificity	Precision	F1-score	G-mean
LD	78.47	79.40	77.83	78.94	78.76	78.61
	(2.16)	(1.99)	(2.50)	(2.29)	(1.60)	(2.23)
LR	79.43	80.38	78.75	79.50	78.99	79.56
	(2.02)	(2.56)	(2.19)	(1.95)	(2.18)	(1.75)
SRC	80.23	80.22	79.26	79.31	79.47	79.74
	(1.79)	(2.53)	(2.30)	(2.54)	(1.39)	(2.31)
LC-KSVD	81.72	82.22	80.59	81.19	81.05	81.40
	(1.31)	(2.34)	(2.41)	(2.22)	(1.35)	(2.40)
FDDL	82.26	83.12	80.87	82.53	82.39	81.98
	(2.20)	(2.37)	(1.42)	(2.56)	(2.44)	(1.84)
SRDML	84.90	85.66	83.86	85.27	85.11	84.76
	(2.27)	(2.49)	(1.80)	(2.83)	(2.13)	(2.12)
LMLS-SRC	**87.90**	**89.25**	**86.53**	**88.71**	**88.44**	**87.88**
	(1.81)	(2.02)	(2.04)	(1.74)	(1.81)	(2.27)

(3) Four-class classification tasks. The main goal of this work is to classify brain MRI images into four classes: very mild AD, non AD, mild AD, and moderate AD. Similar to the three-class classification task described, we randomly selected 1,000 MRI images each from non AD class images and very mild AD, respectively, and used data augmentation to increase the moderate dementia dataset to 520 MRI images. The number of images in the four categories of very mild AD, non AD, mild AD, and moderate AD are 1,000, 1,000, 700, and 520, respectively. The comparison training and test results in four-class classification task are shown in Tables 5, 6, respectively.

**TABLE 5:** The comparative training results (with standard deviation) in four-class classification task.

Algorithms	Accuracy	Sensitivity	Specificity	Precision	F1-score	G-mean
LR	79.70	80.06	78.70	81.49	79.23	79.37
	(1.47)	(2.15)	(2.20)	(2.73)	(1.48)	(2.10)
LR	80.81	81.71	79.41	80.87	80.40	80.55
	(1.88)	(1.47)	(2.09)	(2.11)	(1.22)	(1.27)
SRC	80.86	82.38	79.92	78.97	80.41	81.14
	(2.02)	(2.29)	(1.84)	(1.37)	(1.62)	(2.05)
LC-KSVD	82.61	84.10	80.92	82.36	83.52	82.50
	(2.16)	(1.58)	(1.55)	(2.02)	(2.32)	(1.59)
FDDL	83.85	84.56	82.70	83.46	84.09	83.63
	(1.56)	(2.80)	(2.29)	(3.09)	(2.07)	(2.53)
SRDML	85.91	86.46	83.28	83.39	84.97	84.85
	(2.05)	(2.63)	(2.34)	(2.16)	(1.55)	(2.48)
LMLS-SRC	**86.58**	**87.64**	**85.93**	**86.93**	**86.21**	**86.78**
	(1.59)	(1.13)	(2.45)	(2.00)	(1.49)	(1.66)

**TABLE 6:** The comparative test results (with standard deviation) in four-class classification task.

Algorithms	Accuracy	Sensitivity	Specificity	Precision	F1-score	G-mean
LD	77.67	79.69	77.51	78.40	77.50	78.59
	(2.22)	(1.51)	(2.15)	(2.52)	(2.08)	(1.80)
LR	78.56	79.60	78.47	78.59	78.23	79.03
	(1.89)	(2.51)	(1.60)	(2.74)	(1.43)	(2.01)
SRC	79.40	79.77	79.06	80.25	79.15	79.41
	(2.13)	(2.33)	(2.68)	(1.44)	(2.32)	(2.50)
LC-KSVD	81.25	81.77	81.34	80.87	81.55	81.55
	(2.40)	(2.19)	(1.59)	(2.36)	(2.08)	(1.86)
FDDL	81.45	81.06	80.02	80.67	80.69	80.54
	(1.33)	(2.00)	(2.09)	(2.73)	(1.25)	(2.05)
SRDML	83.13	82.10	82.94	83.56	83.17	82.52
	(2.06)	(2.26)	(2.04)	(1.49)	(1.99)	(2.15)
LMLS-SRC	**85.54**	**86.19**	**84.51**	**86.15**	**85.97**	**85.34**
	(1.59)	(2.03)	(2.12)	(1.63)	(1.06)	(2.07)

We can see that all the comparison algorithms have the highest classification accuracy in the binary classification task (AD and non AD). It shows that these machine learning algorithms have excellent performance in the classification and diagnosis of AD. It is more practical to classify patients, very mild AD, non AD, mild AD, and moderate AD into four classes, and this classification task is more difficult. The classification accuracy of all the comparison algorithms on the four-class task is slightly lower than that on the two-class task. However, the LMLS-SRC algorithm achieves the best results in these tables, indicating that our algorithm has a great improvement in the diagnosis of AD.

In Tables 2, 4, 6, the LMLS-SRC algorithm improves the classification accuracy of the second best algorithm by 2.84, 3.00, and 2.41%, respectively. This shows that the dictionary learned in this study has better reconstruction performance for the samples of same class and better discriminative performance for samples of different classes. KSVD, LC-KSVD, and LMLS-SRC are SRC algorithms. The KSVD and LC-KSVD algorithms only constrain the discriminative ability of the representation coefficients, and do not take into account the large margin between the representation coefficients of different classes. Therefore, the discriminative ability of the learned dictionary obtained by KSVD and LC-KSVD is still weak. The dictionary learned by the LMLS-SRC algorithm in this manuscript is combined with the classification large margin criterion, which directly constrains the intra-class distance and inter-class distance of the representation coefficients. Compared with the other three algorithms, the inter-class differences of the dictionary learned by our algorithm are more discriminative.

### Parameter Analysis

**FIGURE 2:**
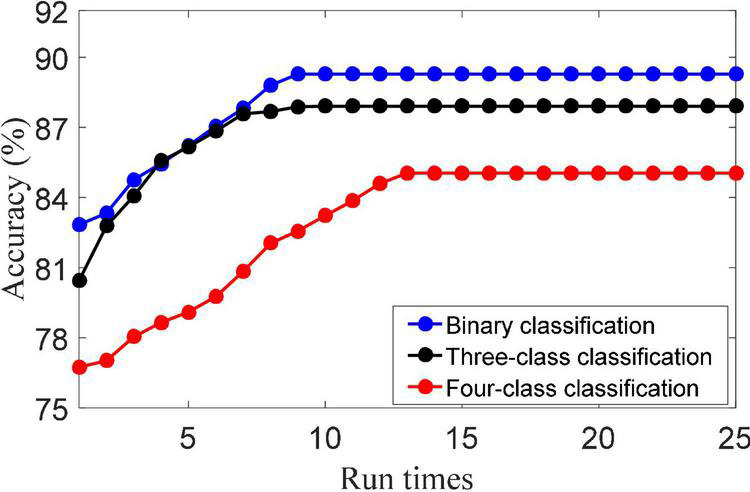
Convergence of the LMLS-SRC algorithm.

**FIGURE 3:**
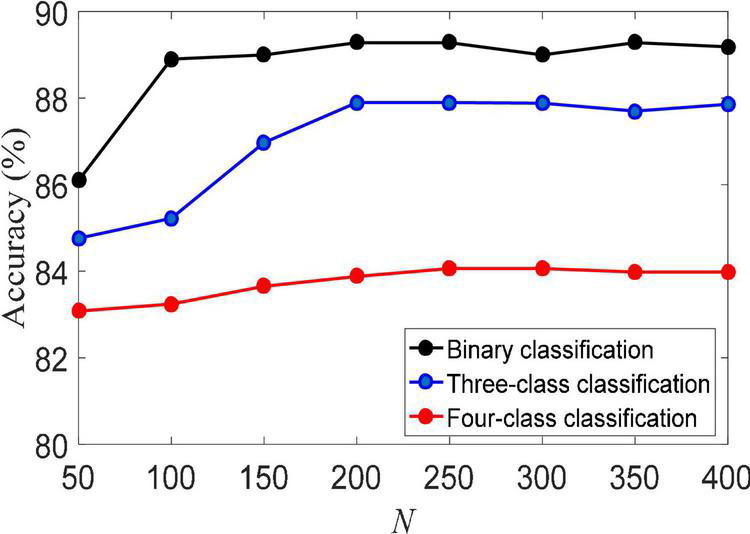
Classification accuracy of local structure preservation sparse representation classifier (LMLS-SRC) under different training sets of each subclass.

**FIGURE 4:**
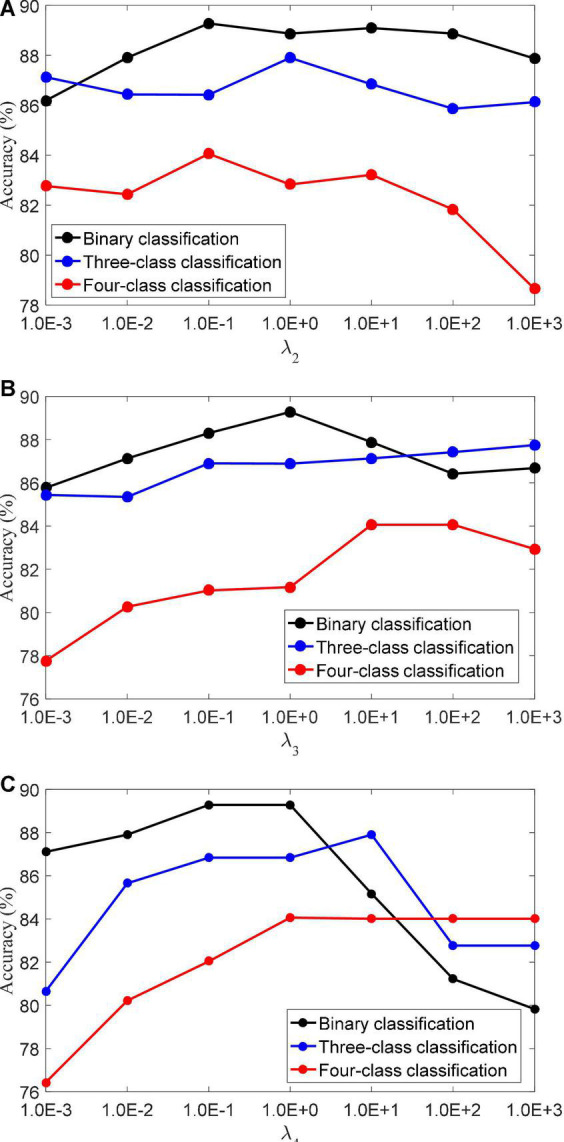
Classification accuracy of LMLS-SRC with different regularization parameters, **(A)**λ_2_, **(B)**λ_3_, and **(C)** λ_4_.

(1) Convergence analysis. The update of {(**D**), (**A**), (**W**)} in the objective function are three convex optimization problems. That is, when other parameters are fixed, the iterative solution of dictionary **D**, representation coefficient **A** and classifier parameter **W** is the convex problem. The solution of dictionary **D** is obtained by Eq. (20). The solution of dictionary **A** is obtained by Eq. (16). The solution of dictionary **W** is obtained by Eq. (22). Figure 2 shows the convergence of the LMLS-SRC algorithm. As shown in Figure 2, it can be seen that the classification accuracy of the LMLS-SRC algorithm tends to be parallel to the X-axis from the 10th iteration. Here, it can be considered that our algorithm converges after 12 iterations.

(2) Training set size. The size of the training set usually directly determines the performance of machine learning algorithms. Figure 3 shows the classification accuracy of the LMLS-SRC algorithm on binary-class, three-class and four-class classification tasks under different training sets of each subclass. The X-axis represents the training sample size *N* of each subclass, *N* = [50, 100,…, 400]. From Figure 3, we can see that the accuracy of LMLS-SRC increases with the increase of training samples. When the training sample size of each subset reaches 200, the performance of the LMLS-SRC algorithm is basically stable, indicating that the LMLS-SRC algorithm can achieve better performance without too many training samples.

(3) Regularization parameters. The LMLS-SRC algorithm has five regularization parameters λ_1_,λ_2_,λ_3_,λ_4_, and λ_5_, and the regularization parameters are all obtained in [1.0E-3…, 1.0E+3]. λ_2_ controls the role of the large margin term. λ_3_ controls the role of the local structure preservation term. λ_*4*_ controls the role of the linear classifier. Figure 4 shows the classification accuracy of the LMLS-SRC algorithm in the binary, three-class and four-class tasks with different λ_2_, λ_3_, and λ_4_, respectively. Figure 4 shows that the performance of the LMLS-SRC algorithm varies greatly with different λ_2_, λ_3_, and λ_4_, while fixing the other parameters. Therefore, it is reasonable to use a grid search strategy to optimize the regularization parameters.

## Conclusion

With the acceleration of the global aging trend, one of the problems brought about is the rapid increase in the number of AD patients. The pathogenesis and effective treatment of AD are still unclear at present. Early detection, classification, and prediction of AD, and targeted care and treatment of patients on this basis can delay the progression of AD. Machine learning algorithms that can automatically extract information and complete inference have good application prospects in AD classification and prediction. Therefore, this manuscript conducts research based on the application of SRC algorithm in AD classification. The research content mainly includes two aspects: model construction and model performance evaluation. The proposed LMLS-SRC algorithm introduces the large margin term and local constraint term in the traditional SRC model, and obtains the dictionary and representation coefficients with discriminative ability while maintaining the data manifold structure. The effectiveness of the LMLS-SRC algorithm is validated on the KAGGLE Alzheimer’s dataset.

Although the LMLS-SRC algorithm shows the advantages compared with some excellent algorithms, there are still some problems to be solved. In the future, we will mainly focus on the following aspects: (1) The LMLS-SRC algorithm belongs to the shallow model. How to design the deep model of the sparse representation algorithm needs to be further studied. (2) In this manuscript, brain MRI images are used as the basic data to study the application of AD classification. Multimodal data can provide richer information, and how to extract AD-related features from multimodal data can be studied in the future. (3) This manuscript uses the volume features extracted by using FSL tool. Extracting various features for AD classification can be done in the next future. (4) In practical applications, image classification often encounters small samples or even a single training sample, and traditional SRC algorithms cannot effectively handle such situations. How to deal with the single training sample is the work to be further studied in the future.

## Data Availability Statement

Publicly available datasets were analyzed in this study. This data can be found here: https://www.kaggle.com/datasets/tourist55/alzheimers-dataset-4-class-of-images.

## Ethics Statement

Ethical review and approval was not required for the study on human participants in accordance with the local legislation and institutional requirements. The patients/participants provided their written informed consent to participate in this study.

## Author Contributions

RL conceived and developed the theoretical framework of the manuscript. All authors carried out experiment and data process, drafted the manuscript, and approved the submitted version.

## Conflict of Interest

The authors declare that the research was conducted in the absence of any commercial or financial relationships that could be construed as a potential conflict of interest.

## Publisher’s Note

All claims expressed in this article are solely those of the authors and do not necessarily represent those of their affiliated organizations, or those of the publisher, the editors and the reviewers. Any product that may be evaluated in this article, or claim that may be made by its manufacturer, is not guaranteed or endorsed by the publisher.
